# Draft Genome Sequence of *Bacillus* sp. Strain BSC154, Isolated from Biological Soil Crust of Moab, Utah

**DOI:** 10.1128/genomeA.01198-14

**Published:** 2014-11-13

**Authors:** Alexis C. Bailey, Matthew Kellom, Amisha T. Poret-Peterson, Kathryn Noonan, Hilairy E. Hartnett, Jason Raymond

**Affiliations:** a School of Earth and Space Exploration, Arizona State University, Tempe, Arizona, USA; b Department of Chemistry and Biochemistry, Arizona State University, Tempe, Arizona, USA

## Abstract

*Bacillus* sp. BSC154 was isolated from a biological soil crust near Moab, Utah. The strain appears to be capable of chemotaxis and biofilm production. The BSC154 genome contains iron siderophore production, nitrate reduction, mixed acid-butanediol fermentation, and assimilatory and dissimilatory sulfate metabolism pathways.

## GENOME ANNOUNCEMENT

*Bacillus* sp. strain BSC154 was collected in an environmental sample from the Green Butte site near Moab, Utah (N38°42′56.2″, W109°41′32″) in May 2009 from a biological soil crust (BSC) (1). BSCs are microbial communities that form in arid and semiarid environments and supply bioavailable carbon (2), nitrogen (3), and metal-containing compounds (4) to their surroundings via carbon and nitrogen fixation as well as siderophore synthesis (5; K. Noonan, A. T. Poret-Peterson, R. M. Potrafka, A. D. Anbar, F. Garcia-Pichel, and H. E. Hartnett, unpublished data). Here we present the draft genome of *Bacillus* sp. BSC154, a sulfate-metabolizing, siderophore-producing member of *Firmicutes*.

BSC154 was cultured and isolated on BG-11 agar plates modified to detect the production of siderophores (6). DNA was extracted from the isolate using the Ultra-Clean Soil DNA Extraction kit, prepped for sequencing using the Illumina TruSeq DNA HT sample prep kit, and sequenced on 21 June 2014 at the Genomics Core of the Biodesign Institute at Arizona State University using the Illumina MiSeq (Illumina RTA 1/18/54): 843,562 300-bp paired-end reads were generated, resulting in 262.24 Mb of raw sequence data, and 190,562 rho *N*_50_ unitigs with a mean length of 371 bp and remaining reads were assembled into 19 gapless scaffolds using the Celera Whole-Genome Shotgun Assembler version 8.1 for a total genome length of 4,028,151 bp (50× coverage) (7). The scaffolds were screened for DNA contaminants and annotated by the NCBI Prokaryotic Genome Annotation Pipeline, which identified 4,032 genes (8). 16S ribosomal analysis showed 99% identity to the 16S sequence of *Bacillus subtilis* strain IAM 12118 from the NCBI 16S Ribosomal RNA Sequences database using BLAST (9). The G+C content was 43.72%, which is consistent with the *Bacillus* genus (10).

Based on the genome of BSC154, it appears to be a heterotrophic member of the BSC microbial community with a complete TCA cycle and terminal cytochrome oxidases that metabolizes carbon compounds produced by other BSC microbes. BSC154 contains the assimilatory nitrogen-compound reduction enzymes nitrate reductase (NasAB) and nitrite reductase (NirBD), the dissimilatory nitrate reduction enzymes nitrate reductase (NarGHIJ), as well as transcriptional regulators Fnr and Rrf2 (11, 12). Being capable of nitrate reduction, BSC154 is a facultative anaerobe also capable of mixed acid-butanediol fermentation, similar to *B. subtilis* strains (11). BSC154 also appears to have complete pathways for assimilatory sulfate reduction and dissimilatory sulfate reduction and oxidation. In addition to sulfate metabolism, BSC154 acquires iron via siderophore production and hydrolysis, containing the pathway for the synthesis of enterobactin as well as iron siderophore ABC transporter permease and enterobactin esterase. BSC154 has genes responsible for chemotaxis, flagellar motility, and biofilm synthesis, which facilitate movement toward and adhesion to areas of favorable nutrient conditions.

### Nucleotide sequence accession numbers.

This whole-genome shotgun project has been deposited at DDBJ/EMBL/GenBank under accession number JPWY00000000. The version described in this paper is version JPWY01000000.
